# *In Vivo* Inhibition of c-MYC in Myeloid Cells Impairs Tumor-Associated Macrophage Maturation and Pro-Tumoral Activities

**DOI:** 10.1371/journal.pone.0045399

**Published:** 2012-09-20

**Authors:** Oscar M. Pello, Raphael Chèvre, Damya Laoui, Alba De Juan, Fidel Lolo, María Jesús Andrés-Manzano, Manuel Serrano, Jo A. Van Ginderachter, Vicente Andrés

**Affiliations:** 1 Laboratory of Molecular and Genetic Cardiovascular Pathophysiology, Department of Epidemiology, Atherothrombosis and Imaging, Centro Nacional de Investigaciones Cardiovasculares Carlos III (CNIC), Madrid, Spain; 2 Laboratory of Cellular and Molecular Immunology, Vrije Universiteit Brussel. Brussels, Belgium; 3 Myeloid Cell Immunology Laboratory, VIB, Brussels, Belgium; 4 Spanish National Cancer Research Center (CNIO), Madrid, Spain; Center for Cancer Research, National Cancer Institute, United States of America

## Abstract

Although tumor-associated macrophages (TAMs) are involved in tumor growth and metastasis, the mechanisms controlling their pro-tumoral activities remain largely unknown. The transcription factor c-MYC has been recently shown to regulate *in vitro* human macrophage polarization and be expressed in macrophages infiltrating human tumors. In this study, we exploited the predominant expression of LysM in myeloid cells to generate *c-Myc^fl/fl^ LysM^cre/+^* mice, which lack *c-Myc* in macrophages, to investigate the role of macrophage c-MYC expression in cancer. Under steady-state conditions, immune system parameters *in c-Myc^fl/fl^ LysM^cre/+^* mice appeared normal, including the abundance of different subsets of bone marrow hematopoietic stem cells, precursors and circulating cells, macrophage density, and immune organ structure. In a model of melanoma, however, TAMs lacking *c-Myc* displayed a delay in maturation and showed an attenuation of pro-tumoral functions (e.g., reduced expression of VEGF, MMP9, and HIF1α) that was associated with impaired tissue remodeling and angiogenesis and limited tumor growth in *c-Myc^fl/fl^ LysM^cre/+^* mice. Macrophage *c-Myc* deletion also diminished fibrosarcoma growth. These data identify *c-Myc* as a positive regulator of the pro-tumoral program of TAMs and suggest *c-Myc* inactivation as an attractive target for anti-cancer therapy.

## Introduction

Macrophages, the mature form of peripheral blood monocytes within tissues, are specialized phagocytic cells involved in multiple processes, both in homeostasis and during the immune response triggered by tissue damage or exposure to pathogens [1], [2]. Tumor-associated macrophages (TAMs) produce factors that promote angiogenesis and tumor cell proliferation, remodel tissue, and dampen the immune response to tumors [3], [4]. TAMs consequently contribute to cancer progression and metastasis in animal models and their density has been associated with poor prognosis in a variety of human tumors, including breast, prostate, bladder, lung and cervical carcinoma, glioma, lymphoma, and melanoma [5], [6], [7]. In response to signals from the local microenvironment, macrophages acquire distinct phenotypes that polarize them toward a specific activation state [2], [8], [9]. For example, activation with IFN-γ, alone or in combination with pathogen-derived signals such as LPS, leads to pro-inflammatory or classically-activated macrophages, also referred to as M1 macrophages, which trigger pro-inflammatory type 1 immune responses. Macrophage exposure to other immune signals results in profoundly different functional phenotypes. These include “alternatively activated” or M2 macrophages, which develop as a consequence of IL-4/IL-13 stimulation, and are associated with type 2 immune responses. Moreover, a spectrum of phenotypes related to anti-inflammatory processes, angiogenesis, and macrophage-regulated tissue repair is induced by a variety of stimuli, including TGF-β, immune complexes, glucocorticoids and IL-10 [8], [9], [10]. Macrophages in tumors are confronted with diverse different microenvironments, leading to the presence of TAM subsets with specialized functions [11]. It has been postulated that TAMs have a predominantly wound healing/regulatory phenotype, resembling that of alternatively activated M2 macrophages [12]. Supporting this notion, TAMs exhibit high production of IL-10 and low production of IL-12, thus suggesting a skewing of L-arginine metabolism toward higher consumption by arginase-1 and lower consumption by iNOS, and deficient expression and function of the transcriptions factors NF-κB and C/EBP, leading to impaired iNOS gene expression and NO production [13], [14]. However, recent studies demonstrated that TAMs also express several M1-associated markers, probably reflecting the existence of TAM subpopulations with distinct functions and located in different tumor regions [11].

Despite the profound effects of macrophage polarization and activation on immune/inflammatory responses and tumor biology, the molecular changes involved in rearranging the transcriptional profile that controls the pro-tumoral phenotype of TAMs remain largely unknown. Because deregulated expression of the proto-oncogene *c-Myc* is associated with tumor development in mice and humans, its role in tumor cell biology has been extensively investigated [15]. c-MYC, which heterodimerizes with MAX to activate expression of targets genes containing the E-box sequence CACGTG in their promoter region [16], [17], is also involved in several processes in non-transformed cells, including cell growth and apoptosis/survival [18]. Resting cells normally express low levels of c-MYC, but expression of this immediate/early response gene is dramatically increased upon exposure to growth factors [19], [20], [21]. Moreover, c-MYC plays essential roles in hematopoietic stem cell function and survival and in lymphoid compartment homeostasis [22], [23], [24], [25], [26], [27]. Recent evidence indicates that c-MYC is induced in human macrophages during alternative activation *in vitro*, and controls the expression of around 50% of alternative-specific markers, suggesting a role in macrophage polarization [28]. In addition, immunohistochemical analysis of colon and breast tumor samples has identified *c-*MYC expression in human TAMs [28].

To investigate the role of macrophage *c-Myc* expression in tumor development, we exploited the predominant expression of LysM in myeloid cells [29] to generate *c-Myc^fl/fl^ LysM^cre/+^* mice, which lack *c-Myc* in macrophages. We investigated the development of melanomas and fibrosarcomas in these animals and characterized the properties of c-MYC deficient TAMs. Our results demonstrate the therapeutic potential of *c-Myc* inhibition as a way to curtail the pro-tumoral functions of TAMs and thereby reduce cancer development.

## Materials and Methods

### Mice and Murine Macrophages

All animal procedures conformed to EU Directive 86/609/EEC and Recommendation 2007/526/EC regarding the protection of animals used for experimental and other scientific purposes, enacted under Spanish law 1201/2005. All animal procedures have been approved by The CNIC Research Ethics Committee (Certificate PA-50/11). To generate mice with macrophage *c-Myc* deficiency, we crossed *c-Myc^fl/fl^* mice [30] with *LysM^cre/+^* mice [29] to obtain *c-Myc^fl/fl^ LysM^cre/+^* mice (*Mφ-c-MycKO* mice) and their *c-Myc^fl/fl^* littermates (control). Bone marrow-derived macrophages (BMDMs) were obtained by flushing mouse tibiae and femurs from control and *Mφ-c-MycKO* mice with ice-cold PBS and passing the suspension through a cell strainer with a 70 µm cut-off. Cells (7×10^6^) were seeded in 100×20 mm non-treated cell culture plates in 10 ml RPMI 1640 supplemented with 10% L929-cell conditioned medium as a source of macrophage colony-stimulating factor (M-CSF) (American Type Culture Collection CCL-1™). Cultures were incubated at 37°C, 5% CO_2_ for 7 days to obtain 95%-pure CD11b+ BMDMs. After this, media were discarded and rinsed twice with saline solution.

Peritoneal macrophages were obtained from the peritoneal cavities of mice, plated in cell culture dishes for 2 h and then extensively washed to eliminate non-attached cells.

### B16 Tumor Model and Tumor-cell–conditioned Medium

B16-F10 murine melanoma cells (a gift from Dr. Maria Soengas, CNIO, Madrid) [31] were cultured in DMEM supplemented with 10% FCS, L-glutamine and penicillin/streptomycin. Once grown to 90% confluence, medium was discarded, and flasks were rinsed twice with saline solution. Cells were then incubated with fresh complete medium for 24 h; the tumor-cell–conditioned medium was collected, filtered (0.20 µm) and stored at −20°C. All cell cultures were routinely checked for *mycoplasma* contamination. For *in vivo* assays, mice were inoculated in the flanks with 5×10^5^ B16 melanoma cells previously infected with lentivirus encoding the firefly luciferase gene [32]. JGA 95.1 murine fibrosarcoma cells [33] were cultured in DMEM supplemented with 10% FCS, L-glutamine and penicillin/streptomycin. For *in vivo* assays, 5×10^5^ JGA 95.1 cells were used to inoculate mouse flanks.

### Macrophage-conditioned Medium

BMDMs were incubated with control medium or tumor-conditioned medium for 24–48 h [28], [34]. After this, medium was discarded, culture plates were rinsed twice with saline solution, and fresh medium was added for 24 h to obtain macrophage-conditioned medium. The collected media were analyzed for metalloproteinase activity in zymography assays [35] or used to stimulate primary murine aortic endothelial cells (MAEC; a gift from Dr. Carlos Zaragoza, CNIC, Madrid) [36] in proliferation and wound healing assays. For proliferation assay, MAECS were labeled with 5 µM CSFE (Sigma-Aldrich) for 5 min and then cultured in macrophage-conditioned medium. MAEC proliferation was measured as the reduction of CFSE fluorescence intensity after 24 h using a FACSCanto flow cytometer (BD Biosciences). For wound healing assay, MAECs were grown to confluence in 6-well tissue culture plates. A “wound” was made by scraping with a P200 pipette tip across the center of the monolayer, and macrophage-conditioned medium was added. Cell migration into the denuded area was monitored by time-lapse photomicroscopy and quantified with ImageJ software.

For CD8^+^ T proliferation/suppression assays, 2×10^5^ naive BALB/c splenocytes were labeled with 5 µM CFSE, stimulated with 1 µg/mL anti-CD3 to induce proliferation and cultured in macrophage-conditioned medium. 24 h later CD8^+^ T cells were labeled with anti-CD8-647 [11] and proliferation was measured as the reduction of CFSE fluorescence intensity using a FACSCanto flow cytometer (BD Biosciences).

### Flow Cytometry and Cell Sorting

Cells were analyzed with a FACSCanto flow cytometer and FACSDiva software (BD Biosciences). Viable cells were identified by propidium iodide exclusion. Singlet cells were discerned with a stringent multiparametric gating strategy based on FSC and SSC (pulse width and height). Cells were sorted on a FACSAria flow cytometer (BD Biosciences). The following rat anti-mouse antibodies were used for FACS analysis: Gr1-FITC (53-5931), CD115-PE (12-1152), CD16/32-PE (12-0161), CD117-PE (25-1171), CD34-Alexa 660 (50-0341), Sca1-FITC (11-5981-85), Ly6G-FITC (551460), CD45-PE (12-0454-81) from eBioscense; F4/80-Alexa 647 (MCA497A647), F4/80-Alexa 488 (MCA497A488), MRC1-PE (MCA2235PE) from Serotec; CD11b-PeCy7 (552850), CD8-APC (553035 BD), CD4-Percpcy5.5 (550954), CD19-FITC (553785), SiglecF-PE (552126) from BD Biosciences; CD45-biot (553078), biotin mouse lineage (559971), CD11c-PE (553802), IL4Ra-PE (552509) from BD Pharmingen; MHCII-PercpCy5.5 (107626) from Biolegend; Ly6C-ALexa 647 (MCA2389A647) from BD Biosciences.

### RNA Isolation and Quantitative Real-time PCR (qPCR)

Total RNA was extracted with TRIzol reagent (Invitrogen) and the miRNeasy mini kit (Qiagen), and was screened for purity and concentration in a Nanodrop-1000 Spectrophotometer (Thermo Scientific). First-strand cDNA was synthesized from 2 µg total RNA in a 25 µl reaction mixture using the High Capacity cDNA Achieve kit (Applied Biosystems). The following gene-specific primers for qPCR were designed using Autoprime software (www.autoprime.de):

c-MYC-Forward: 5′GAGCTGTTTGAAGGCTGGATTT3’

c-MYC*-*Reverse: 5′TCCTGTTGGTGAAGTTCACGTT3’

VEGFA-Forward: 5′GGAGATCCTTCGAGGAGCACTT3’

VEGFA-Reverse: 5′GGCGATTTAGCAGCAGATATAAGAA3’

MMP9-Forward: 5′CAAATTCTTCTGGCGTGTGA3’

MMP9-Reverse: 5′CGGTTGAAGCAAAGAAGGAG3’

HIF1α-Forward: 5′TCAACCACAGGACAGTACAGGATGC3’

HIFα-Reverse: 5′CCAGCAAAGTTAAAGCATCAGGTTCC3’

N-MYC-Forward: 5′ CTGAGCTGGTGAAGAACGAGAA3’

N-MYC-Reverse: 5′ CTCGGTGGCCTTTTTCAAGA3’

LDH-Forward: 5′ AGGCTCCCCAGAACAAGATT3’

LDH-Reverse: 5′ TCTCGCCCTTGAGTTTGTCT3’

iNOS-Forward: 5′ GGATCTTCCCAGGCAACCA3’

iNOS-Reverse: 5′ TCCACAACTCGCTCCAAGATT 3′.

Three replicates were performed for each experimental point, and differences were assessed with a two-tailed Student’s *t* test. Results were normalized using the housekeeping gene GAPDH and the ΔΔ cycle threshold method, and are expressed in arbitrary units.

### Immunohistochemistry, Confocal Microscopy and Imaris Analysis

Macrophage tumor infiltration was examined by immunohistochemistry performed by a researcher blinded to genotype. After sacrifice, tumor specimens were either frozen immediately after surgical removal or fixed in buffered formalin and embedded in paraffin. Macrophages were detected with a rat anti-CD68 monoclonal antibody (1/200 dilution, MCA1957, Serotec) followed by anti-rat Alexa488-conjugated secondary antibodies (A11006, A-11034, Invitrogen). c-MYC expression was analyzed with rabbit anti-c-MYC polyclonal antibody (06-340, Millipore) followed by Alexa635-conjugated anti-rabbit IgG antibody (A21429, Invitrogen).

Macrophage proliferation in tumors was assessed by double immunofluorescence with rabbit anti-Ki67 antibody (prediluted, Clone SP6, MasterDiagnostica) together with rat anti-CD68 followed by Alexa635-conjugated anti-rabbit IgG antibody (A21429 Invitrogen) and anti-rat Alexa488-conjugated secondary antibodies (A11006, A-11034, Invitrogen). Macrophage apoptosis was measured with the In situ Cell Death detection kit (Roche).

Cell nuclei were stained with TOPRO-3 (T3605, Invitrogen). Slides were mounted with Slow-Fade Gold Antifade reagent (S36936, Invitrogen) and images were acquired on a Leica TCS/SP2 confocal microscope fitted with a 40X oil immersion objective. Vessel coverage in tumors was assessed by quantification of rabbit anti-CD31 (ab28364, Abcam) staining with Imaris software in at least 3 tumors per genotype corrected for the selected area.

### *In vivo* Microscopy (IVIS) and Fluorescence Molecular Tomography (FMT)

D-Luciferin (Sigma-Aldrich) was injected i.p. 15 min before IVIS. IntegrinSense 750 and MMPSense 680 probes (Visen) were injected i.v. 18 h before FMT. Mice were anesthetized with isoflurane/oxygen mixture and then placed in an imaging cassette (PerkinElmer/VisEn Medical). After positioning the cassette in the FMT1500 imaging system (PerkinElmer/VisEn Medical), reflectance images were captured in white light and fluorescence (2D planar). For 3D imaging, a field around the tumor was selected, and the tomographic scan was carried out. The scan data were analyzed with the reconstruction software provided by the manufacturer (PerkinElmer/VisEn Medical). For tomographic data analysis, three-dimensional regions of interest (ROI) were drawn around the tumor, and the total amount (in picomoles) of fluorochrome was calculated with TrueQuant software (PerkinElmer/VisEn Medical) using previously generated standards of the appropriate dye.

### Statistical Analysis

Results are expressed as mean±SD and were analyzed using GraphPad-Prism (GraphPad Software, LaJolla, CA). Differences were considered statistically significant at p<0.05, as determined by unpaired 2-sided Student’s *t* test (experiments with two groups) or two-way ANOVA followed by Bonferroni’s test (experiments with more than two groups).

## Results

### Generation of *Mφ-c-Myc-KO* Mice

**Figure 1:**
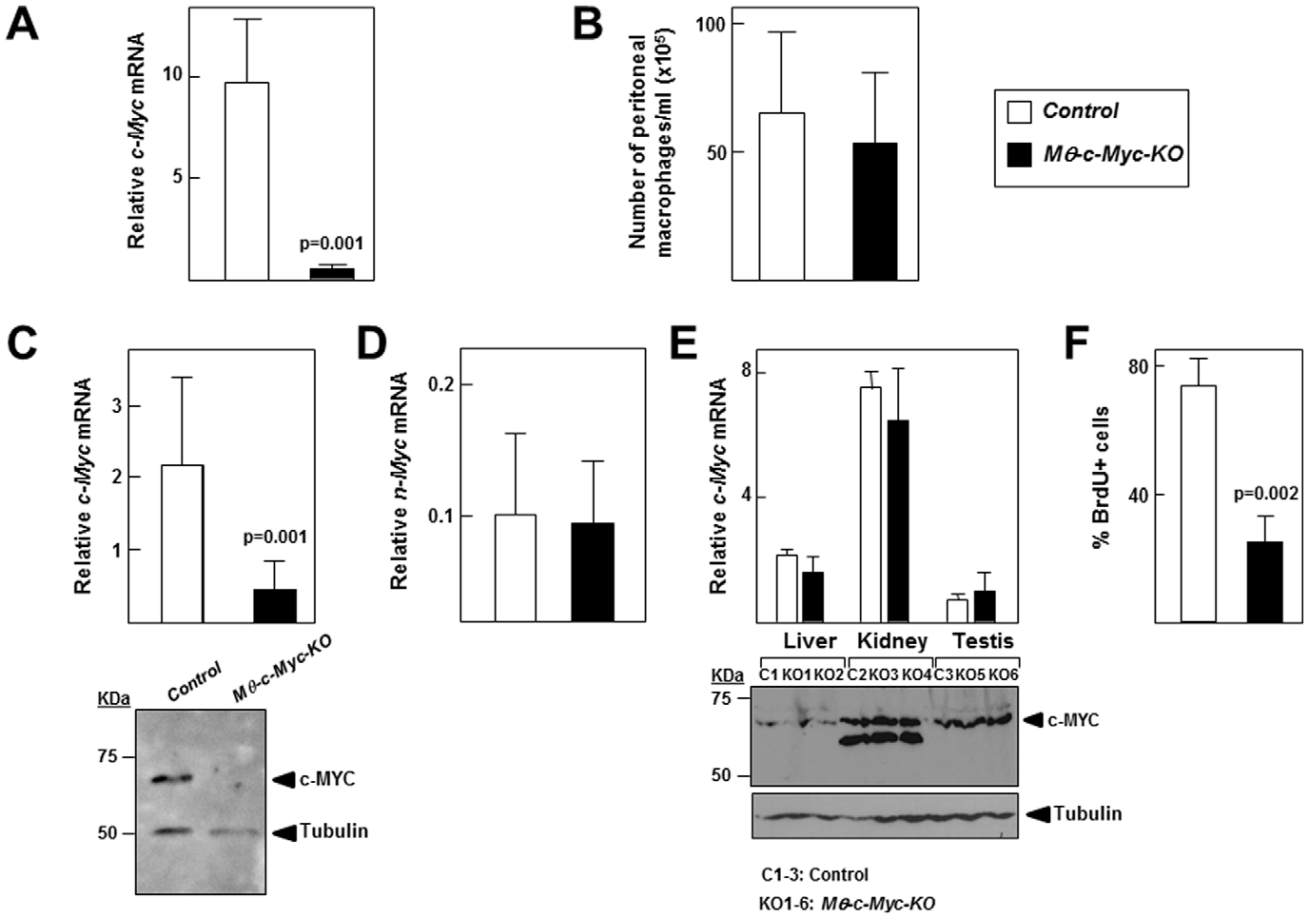
*Mφ-c-Myc-KO* mice exhibit reduced c-MYC expression in macrophages. (**A**) *c-M*yc mRNA expression determined by qPCR in peritoneal macrophages from control and *Mφ-c-Myc-KO* mice (n = 7 per genotype). (**B**) Total numbers of peritoneal macrophages isolated from control and *Mφ-c-Myc-KO* mice after washing the peritoneal cavity with 3 ml PBS (n = 8 mice per genotype). (**C**) Expression levels of *c-Myc* mRNA (top, n = 6 per genotype) and c-MYC protein (bottom, representative blot) in BMDMs. (**D**) *n-M*yc mRNA expression determined by qPCR in BMDMs from control and *Mφ-c-Myc-KO* mice (n = 4 per genotype). (**E**) Expression levels of *c-Myc* mRNA (top) and protein analyzed by western blot (bottom) in liver, kidney and testis (n = 3 control, n = 6 *Mφ-c-Myc-KO*). (**F**) BMDM proliferation assessed by 5′-bromodeoxyuridine (BrdU) incorporation (n = 3 per genotype).

Previous studies demonstrated the predominant expression of *LysM* in the myeloid lineage [29]. Using this system, we crossed *c-Myc^fl/fl^* mice [30] with *LysM^cre/+^* [29] mice to generate *Mφ-c-Myc-KO* mice, in which *c-Myc* is deleted in macrophages, and control *c-Myc^fl/fl^* littermates, in which *c-Myc* expression is intact. qPCR confirmed significantly reduced levels of *c-Myc* in naïve peritoneal macrophages from *Mφ-c-Myc-KO* mice compared with controls (**Fig. 1A**), with no effect on total cell number (**Fig. 1B**) or morphological characteristics in culture (**data not shown**). We also found markedly lower *c-Myc* mRNA and c-MYC protein levels in BMDMs obtained from *Mφ-c-Myc-KO* mice (70–100% reduction across all analyzed mice) (**Fig. 1C**), whereas expression of *n-Myc* **(** **Fig. 1D** **)** and the macrophage-specific marker F4/80 were unaffected (data not shown). In contrast, *c-Myc* mRNA and protein expression levels were normal in liver, kidney and testis (**Fig. 1E**), non-immune tissues in which macrophages represent a minor fraction. We next analyzed the proliferative capacity of *Mφ-c-Myc-KO* BMDMs upon stimulation with M-CSF [37]. Consistent with previous reports that blockade of BMDM proliferation correlates with decreased *c-Myc* expression [38], [39], BrdU incorporation assays showed reduced *de novo* DNA synthesis in *c-Myc-*deficient BMDMs (**Fig. 1F**).

### *Mφ-c-Myc-KO* Mice do not Exhibit Immune Alterations Under Steady-state Conditions

**Figure 2:**
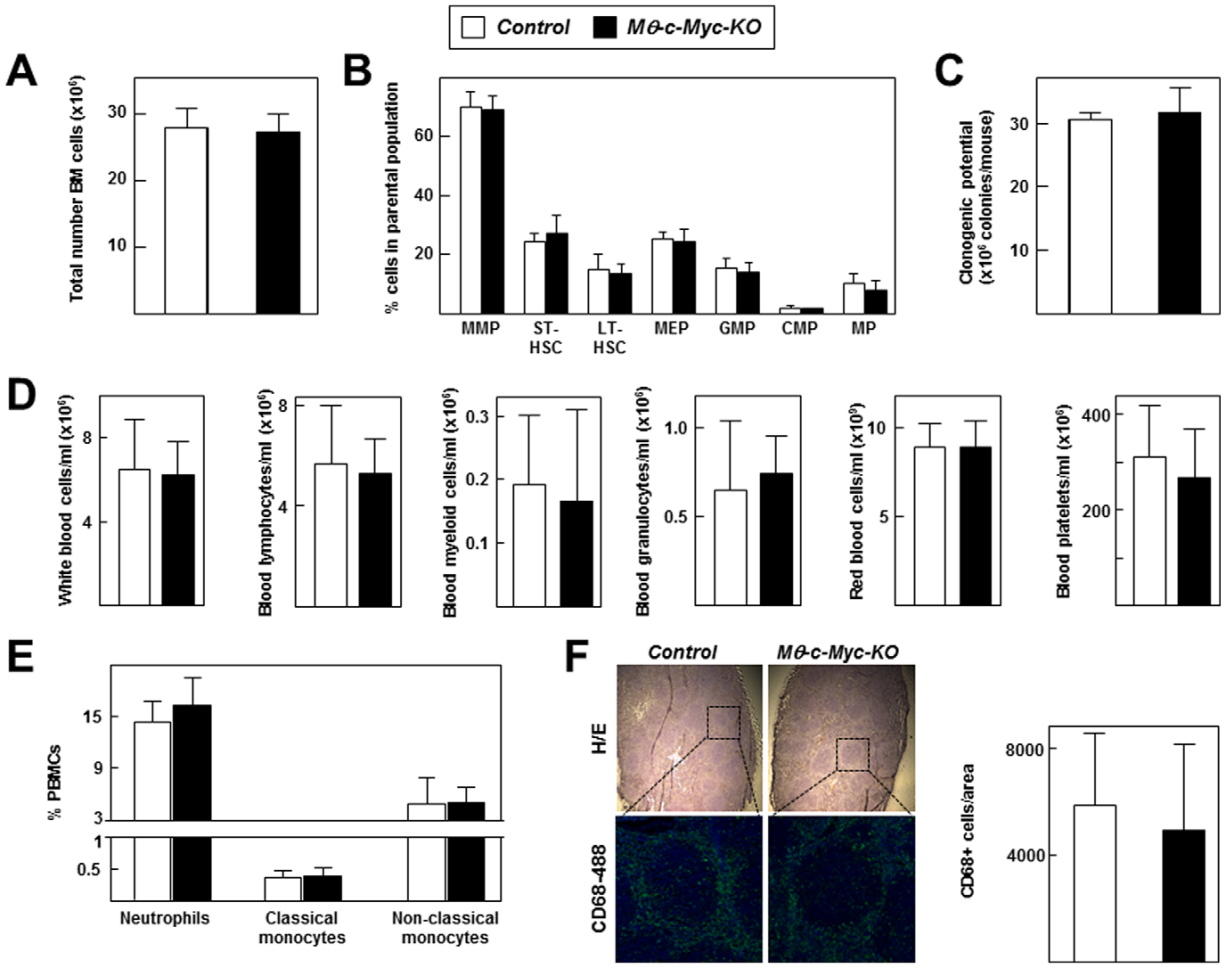
Characterization of the immune system of *Mφ-c-Myc-KO* mice under steady-state conditions. All parameters were analysed in 6 mice per genotype. (**A**) Total numbers of bone marrow cells isolated from mice after flushing femurs and tibias with PBS. (**B**) Flow cytometry analysis of bone marrow precursors. MMPs, LT-HSCs, ST-HSCs were analyzed by double immunostaining for Flk2 and Cd90 within the parental Lin^−^ Sca1^+^ c-Kit^+^ (LSK) population. MEPs, GMPs and CMs were analyzed by double immunostaining for CD16 and CD34 within the parental LSK population. MPs were detected by staining for CD115^+^ within the parental Lin^−^ population. (**C**) Clonogenic activity of total bone marrow cells. (**D**) Blood hemogram analysis for the indicated cell types. (**E**) Flow cytometry of blood cells doubly stained for Gr-1 and CD115 to identify neutrophils (Gr-1^high^CD115^−^), classical monocytes (Gr-1^high^CD115^+^) and non-classical monocytes (Gr-1^−^CD115^+^). (**F**) Spleen sections were either stained with hematoxilin/eosin or analyzed by confocal microscopy to visualize macrophage infiltration (CD68+ cells) and nuclei (DAPI staining). Representative images are shown, with higher magnification for CD68 immunofluorescence showing macrophage-rich red pulp surrounding a spleenic follicle. The graph shows the quantification of CD68^+^ macrophages within the spleen using Imaris software (n = 3 tumors per genotype).

In adult mammals, hematopoiesis takes place within the marrow of large bones, and *c-*MYC is important for establishing the correct balance between self-renewal and differentiation of hematopoietic stem cells [22], [25]. To test whether *c-Myc* inactivation affects hematopoiesis in our model, we compared BM from control and *Mφ-c-Myc-KO* mice. Total BM cell counts were undistinguishable between the two genotypes (**Fig. 2A**). Likewise, flow cytometry analysis of distinct BM hematopoietic precursors by double Flk2/CD90 immunostaining within the parental Lin^−^ Sca1^+^ c-Kit^+^ population (LSK) [40] revealed no differences in the levels of multipotent progenitors (MPPs), long-term hematopoietic stem cells (LT-HSCs) or short-term hematopoietic stem cells (ST-HSCs) **(** **Fig. 2B** **)**. Double immunostaining for CD16 and CD34 in the LSK population [41] also showed no differences in the levels of multiple erythroid progenitors (MEPs), granulocyte-macrophage progenitors (GMPs) or common myeloid progenitors (CMPs). Likewise, staining of the Lin^−^ population for CD115^+^ [42] revealed no differences in the numbers of macrophage progenitors (MPs) (**Fig. 2B**). *Ex vivo* functional analysis of BM from both genotypes confirmed similar clonogenic potential of myeloid precursors (**Fig. 2C**).

Hemogram analysis of circulating BM-derived cells showed no significant differences between *Mφ-c-Myc-KO* and control mice in counts of total white blood cells, lymphocytes, myeloid cells, granulocytes, red blood cells and platelets **(** **Fig. 2D**). The phagocytic compartment was analyzed in further detail by flow cytometry staining for Gr-1 and CD115 to identify neutrophils, classical monocytes, and non-classical monocytes [43]. These studies revealed no significant differences between the peripheral blood profiles of *Mφ-c-Myc-KO* and control mice (**Fig. 2E**).

Since macrophages are mature forms of peripheral blood monocytes that infiltrate tissues [44], we studied their content in immune organs under steady-state conditions. The two genotypes showed similar tissue architecture and number and distribution of macrophages in spleen (**Fig. 2F**) and in thymus and lymph nodes (**data not shown**).

### Reduced *c-Myc* Expression in Macrophages Attenuates B16 Melanoma Tumor Growth without Affecting Proliferation or Survival of TAMs

**Figure 3:**
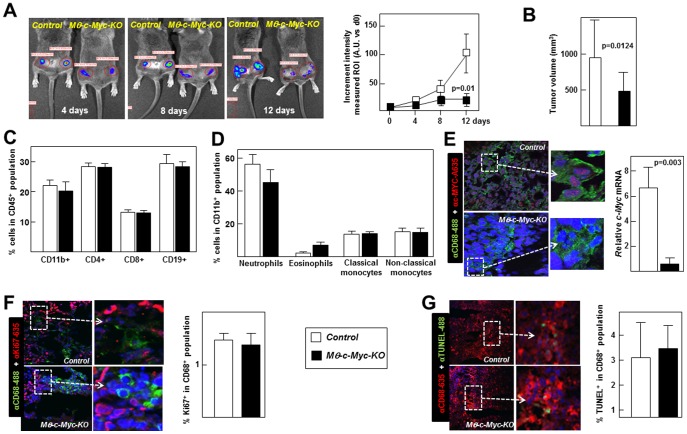
*Mφ-c-Myc-KO* mice are resistant to growth of tumors derived from injected B16 melanoma cells. (**A**) B16 melanoma cells expressing firefly luciferase were injected subcutaneously into both flanks of mice, and tumor growth was monitored over two weeks post-grafting with the Xenogen IVIS 100 Bioluminescence *in vivo* imaging system (n = 6–8 tumors per genotype). (**B**) Tumor volume after sacrifice at two weeks post-injection (n = 16 tumors per genotype). (**C**) Flow cytometry analysis for circulating CD11b^+^ myeloid cells, CD4^+^ T lymphocytes, CD8^+^ T lymphocytes and CD19^+^ B lymphocytes in mice two weeks after tumor injection (n = 7, control; n = 8 *Mφ-c-Myc-KO*). (**D**) Flow cytometry detection of circulating myeloid cells two weeks after tumor injection (n = 7, control; n = 8, *Mφ-c-MYC-KO*) (**E**) Representative confocal images showing double immunofluorescence to visualize macrophages (CD68^+^ cells) and c-MYC expression in tumors, and quantification of *c-Myc* mRNA in TAMs isolated by sorting tumor cell suspensions (n = 3 mice per genotype; two tumors pooled per mouse). (**F**) Representative confocal images and quantification of proliferating TAMs (CD68+Ki67+) (n = 6 tumors per genotype). (**G**) Representative confocal images and quantification of apoptotic TAMs (CD68+TUNEL+) (n = 6 tumors per genotype).

To investigate the effect of macrophage *c-Myc* deletion on cancer, we used the B16 melanoma model, in which tumor growth is critically dependent on the presence of TAMs [45]. B16 melanoma cells (5×10^5^) previously infected with lentivirus carrying the firefly luciferase gene [32] were injected subcutaneously in the flanks of control and *Mφ-c-Myc-KO* mice (2 tumors/mouse). Bioluminescence *in vivo* imaging at successive intervals after grafting revealed a significant upregulation of luciferase intensity in control tumors at 12 days post-grafting that was absent in tumors from *Mφ-c-Myc-KO* mice **(** **Fig. 3A**). Consistent with these findings, postmortem studies demonstrated a significant reduction in tumor volume in *Mφ-c-Myc-KO* mice compared with control animals **(** **Fig. 3B**), and these differences occurred without alterations in circulating B and T lymphocytes or CD11b^+^ cells (neutrophils, eosinophils, and classical and non-classical monocytes) (**Fig. 3C, D**). As expected, examination of excised tumors by double immunofluorescence revealed c-MYC expression in TAMs from control mice, whereas it was undetectable in TAMs from *Mφ-c-Myc-KO* mice (**Fig. 3E** **, left**). Severely impaired *c-Myc* expression in cells from *Mφ-c-Myc-KO* mice was confirmed by qPCR analysis of sorted TAMs **(** **Fig. 3E** **, right)**. We also performed double immunofluorescence experiments in TAMs to evaluate proliferation (CD68^+^/Ki67^+^ co-expression, **Fig. 3F**) and apoptosis (CD68^+^/TUNEL^+^ co-expression, **Fig. 3G**). These studies demonstrated no differences in proliferative activity (CD68^+^/Ki67^+^: 1.5±0.5% in control versus 1.4±0.6 in *Mφ-c-Myc-KO*) (**Fig. 3F**) or apoptotic cell death (CD68^+^/TUNEL^+^: 3.1±1.5% in control versus 3.6±0.9 in *Mφ-c-Myc-KO*) (**Fig. 3G**). Notably, we did observe differences between genotypes in the Ki67 signal from CD68 negative cells, which are mostly tumor cells (CD68^−/^Ki67^+^: 49±5.7% in control versus 30.6±3.3 in *Mφ-c-Myc-KO*).

### Reduced *c-Myc* Expression in Macrophages Alters Tumor T Cell Infiltration and TAM Phenotype

**Figure 4:**
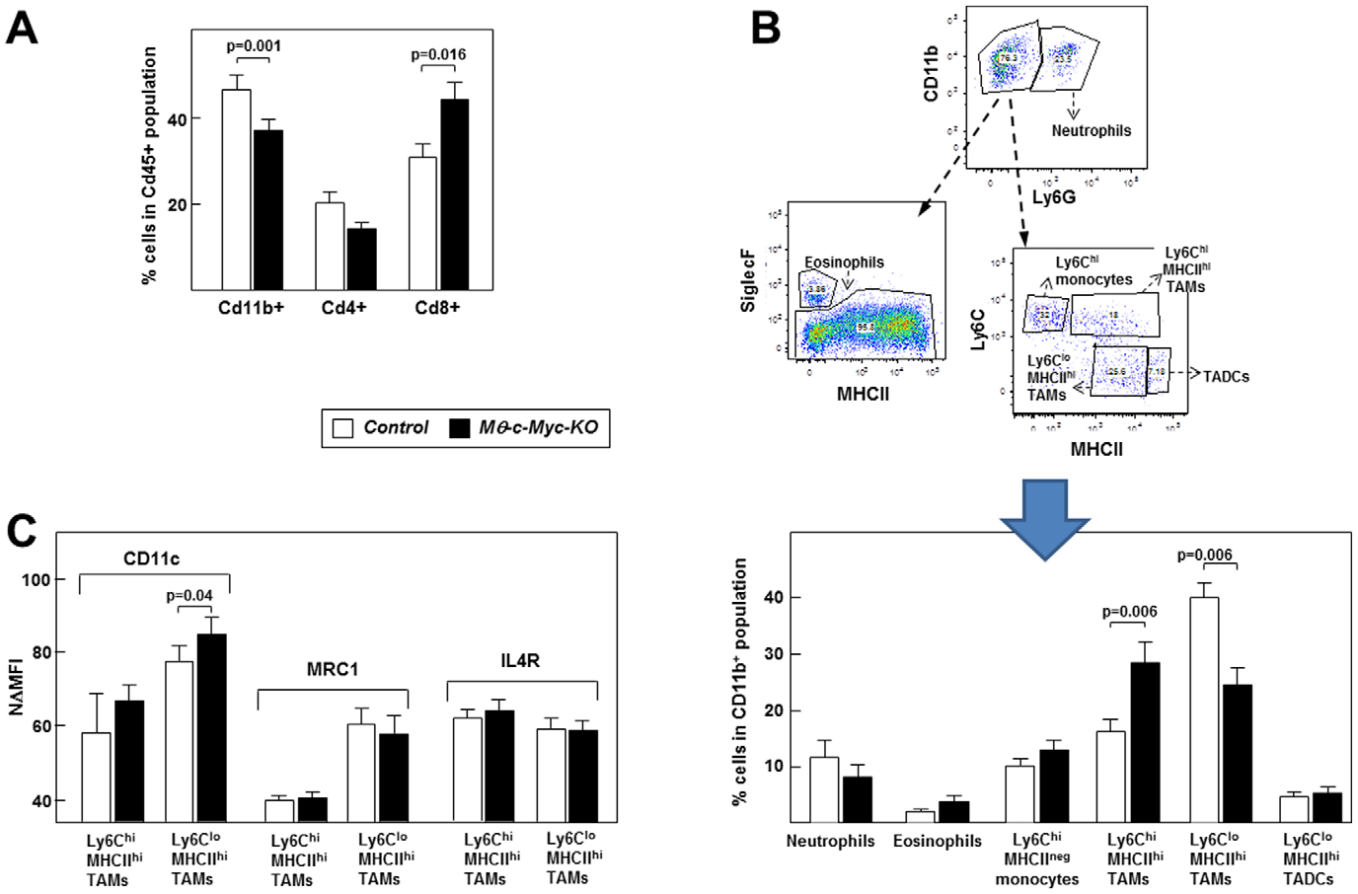
*c-Myc* deletion in macrophages affects T cell infiltration and the TAM phenotype in tumors. Flow cytometry analysis of single-cell suspensions prepared from tumors obtained 17–25 days after injection of B16 cells (n = 11 control tumors; n = 9 *Mφ-c-Myc-KO* tumors). (**A**) CD45^+^ immune cells were gated to identify the proportion of CD11b^+^ myeloid cells and CD4^+^ and CD8^+^ T lymphocytes. (**B**) The strategy shown on the left was used to quantify distinct myeloid cell subsets in gated Cd11b^+^ Ly6G- cells. (n = 13 control tumors; n = 9 *Mφ-c-Myc-KO* tumors) TADCs: tumor-associated dendritic cells. (**C**) Analysis of CD11c, MRC1 and IL4R in TAMs (n = 6 control tumors; n = 7 *Mφ-c-Myc-KO* tumors). Results are represented as normalized ΔMFI (MFIab-MFIisotipe/MFIisotipe*100).

After sacrifice, tumors were dissociated and their immune infiltrate analyzed by flow cytometry. Numbers of CD19^+^ B lymphocytes were low in both genotypes and differences in the percentage of CD45^+^ cells and CD4^+^ T lymphocytes did not reach statistical significance **(** **Fig. 4A** **and data not shown)**. In contrast, tumors in *Mφ-c-Myc-KO* mice contained significantly lower numbers of myeloid CD11b+ cells and higher numbers of CD8^+^ T cells (**Fig. 4A**). A more detailed analysis of tumor-associated myeloid cells showed no significant differences in the relative abundance of neutrophils, eosinophils, Ly6C^high^MHCII^neg^ monocytes or dendritic cells between genotypes **(** **Fig. 4B** **)**. However, TAM subsets differed significantly, with more Ly6C^high^MHCII^high^ TAMs and less Ly6C^low^MHCII^high^ TAMs in tumors from *Mφ-c-Myc-KO* mice compared with controls **(** **Fig. 4B** **)**. Further characterization of the polarization status of TAMs of both genotypes showed similar levels of the M2-like markers MRC1 and IL4R but higher CD11c expression in *Mφ-c-Myc-KO* TAMs compared with controls (**Fig. 4C**).

### Pro-tumoral Activity is Diminished in TAMs with Reduced *c-Myc* Expression

**Figure 5:**
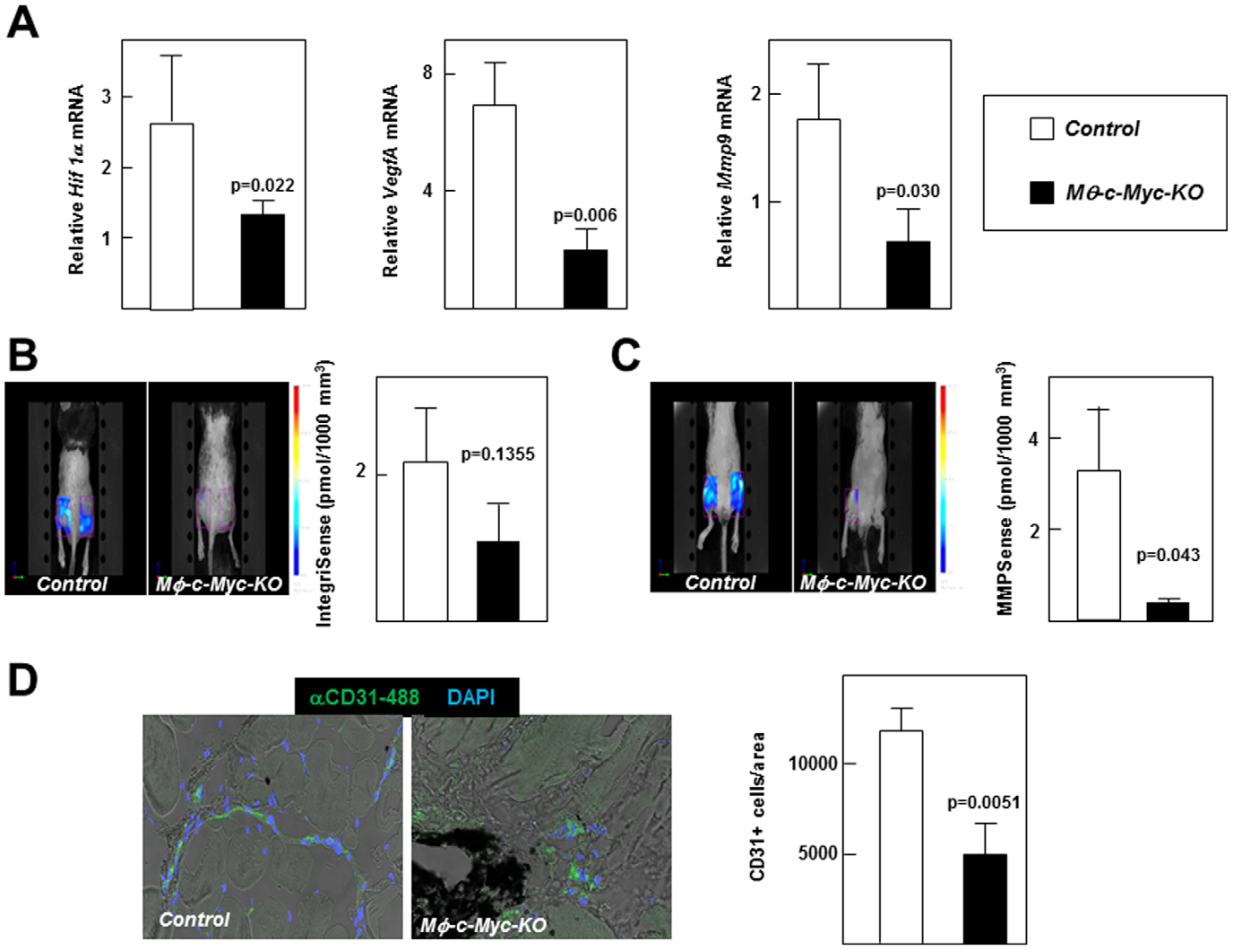
TAMs isolated from *Mφ-c-Myc-KO* mice show reduced pro-tumoral activity. B16 melanoma cells were injected into both flanks of mice, and tumors were analyzed after 2 weeks. (**A**) Levels of pro-tumoral transcripts (*VegfA*, *Mmp9*, and *Hif1α*) in TAMs obtained by sorting single-cell suspensions of tumors (n = 3 mice per genotype; two tumors pooled per mouse). (**B**) Representative FMT images of tumors after injecting IntegrinSense 750 probe to monitor angiogenic activity *in vivo*. The graph shows quantification normalized to a standard volume of 1000 mm^3^ (n = 10 control tumors; n = 12 *Mφ-c-Myc-KO* tumors). (**C**) Representative FMT images of tumors after injecting MMPSense 680 probe to monitor metalloproteinase activity *in vivo*. The graph shows quantification normalized to a standard volume of 1000 mm^3^ (n = 10 control tumors; n = 12 *Mφ-c-Myc-KO* tumors). (**D**) Representative confocal images showing blood vessels (CD31^+^ endothelial cells). The graph shows quantification of CD31^+^ cells per tumor area using Imaris software (n = 3 tumors per genotype).

The distinct proliferative and metastatic capacities of different tumors were recently shown to be associated with the specific pro-tumoral characteristics activities of the TAMs they contain [11]. Moreover, different TAM subsets within a tumor have been suggested to contribute differentially to tumor growth [11]. To investigate the impact of macrophage *c-Myc* deletion on the pro-tumoral activity of TAMs, we sorted B16 melanoma tumor cell suspensions and analyzed by qPCR the expression of three factors required for tissue remodeling and angiogenesis in cancer: hypoxia-inducible factor 1α (HIF1α), vascular endothelial growth factor (VEGFA) and metalloproteinase-9 (MMP9). Expression of these pro-tumor factors was significantly lower in TAMs isolated from *Mφ-c-Myc-KO* mice compared with controls (**Fig. 5A**). Next, we performed non-invasive FMT of tumors from mice injected with IntegrinSense 750 (**Fig. 5B**) and MMPSense 680 (**Fig. 5C**) probes to detect angiogenesis and metalloproteinase activity, respectively [46], [47]. Consistent with lower angiogenic activity and tissue remodeling, intensity of both probes was lower in tumors from *Mφ-c-Myc-KO* mice, reaching statistically-significant differences for MMPSense (**Fig. 5B,C**). Although these results might reflect in part the bigger size of the tumors in control mice, expression of the endothelial-cell–specific marker CD31 per area of tumor was lower in tumors from *Mφ-c-Myc-KO* mice (**Fig. 5D**).

**Figure 6:**
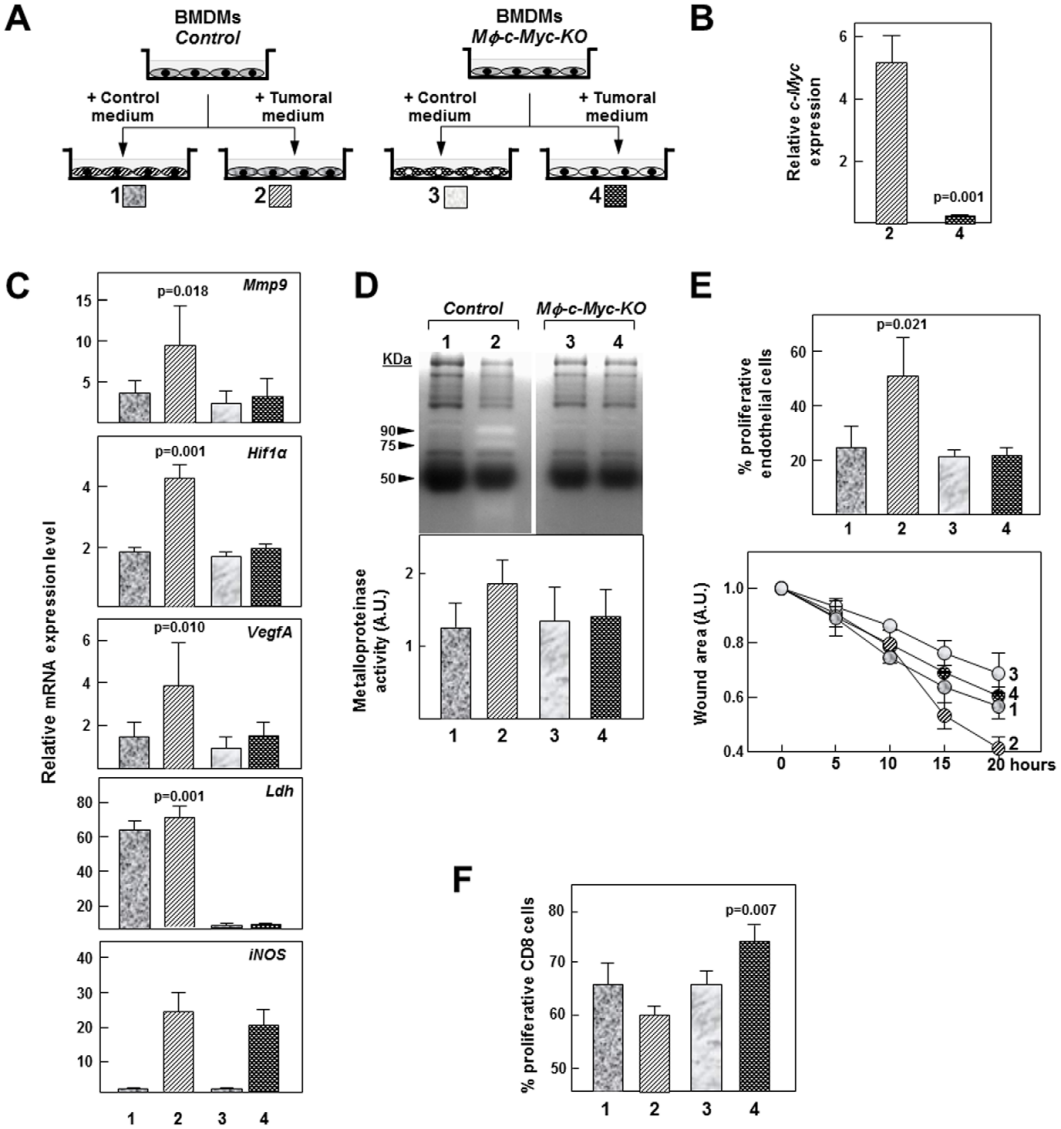
*In vitro* analysis of the pro-tumoral behavior of macrophages from control and *Mφ-c-Myc-KO* mice. (**A**) Strategy for studying pro-tumoral behaviour of macrophages cultured *in vitro* in conditioned medium from control medium or tumor conditioned medium from B16 melanoma cells (n = 3 per genotype) (**B**) Expression levels of *c-Myc* mRNA in control and *Mφ-c-Myc-KO* BMDMs treated with tumor-cell–conditioned medium. (**C**) Transcript levels in BMDMs from both genotypes treated with control or tumor-cell–conditioned medium. (**D**) Top: Representative zymography analysis of metalloproteinase activity in supernatants from BMDMs treated with control or tumor-cell–conditioned medium. Bottom: Quantification of metalloproteinase activity determined by zymography analysis (n = 3 experiments). (**E**) Top: Quantification of endothelial cell proliferation 24 h after stimulation with supernatant from BMDMs treated with control or tumor-cell–conditioned medium. Bottom: Quantification of wound area 24 h after stimulation of scrape-damaged endothelial cultures with supernatant from BMDMs treated with control or tumor-cell–conditioned medium. (**F**) Quantification of CD8-T lymphocyte proliferation 24 h after stimulation with supernatant from BMDMs treated with control or tumor-cell–conditioned medium.

To further investigate the role of *c-*MYC in the pro-tumoral activities of TAMs, we exposed BMDMs to conditioned culture medium from tumor cells **(** **Fig. 6A** **),** an *in vitro* model that allows characterization of cell autonomous effects [28], [34]. qPCR confirmed that control BMDMs exposed to tumor-cell–conditioned medium expressed higher levels of *c-Myc* than *Mφ-c-Myc-KO* cells (**Fig. 6B**). Moreover, in line with our observations in TAMs, tumor-cell–conditioned medium induced *Hif1α*, *VefgA* and *Mmp9* expression in control but not in *Mφ-c-Myc-KO* BMDMs (**Fig. 6C** **)**. Transcript levels of lactate dehydrogenase *(Ldh),* which is a downstream target of c-MYC [48], was strongly reduced in *Mφ-c-Myc-KO* BMDMs **(** **Fig. 6C** **)**. In contrast, induction of the M1-like marker inducible nitric oxide synthase *(iNos)* by tumoral medium was c-MYC independent **(** **Fig. 6C** **)**. The lower *Mmp9* expression in *Mφ-c-Myc-KO* BMDMs correlated with lower metalloproteinase activity, as revealed in zymogram assays (**Fig. 6D**). Moreover, whereas culture supernatants from control BMDMs treated with tumor-cell–conditioned medium promoted endothelial cell proliferation and migration in wound healing assays, wound healing was impaired in response to supernatants from similarly conditioned *Mφ-c-Myc-KO* BMDMs (**Fig. 6E**). Finally, culture supernatants from *Mφ-c-Myc-KO* BMDMs treated with tumor-cell–conditioned medium showed lower ability to reduce CD8^+^ T-lymphocyte proliferation (**Fig. 6F**). Together these results highlight the role of c-MYC as a key positive regulator of the pro-tumoral activity of mature TAMs.

### Reduced *c-Myc* Expression in Macrophages Attenuates Fibrosarcoma Tumor Growth

**Figure 7:**
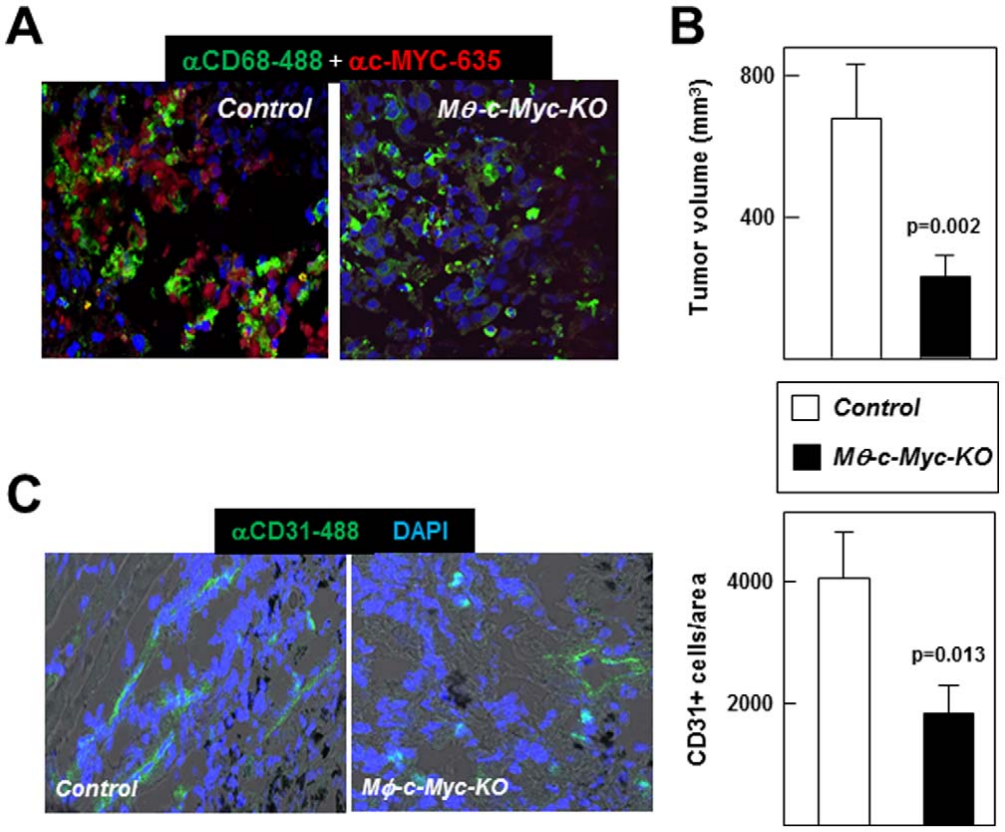
*Mφ-c-Myc-KO* mice are resistant to fibrosarcoma growth and tumor angiogenesis. JGA 95.1 murine fibrosarcoma cells were injected into both flanks of mice, and tumors were analyzed after 2 weeks. (**A**) Representative confocal double immunofluorescence images showing macrophages (CD68^+^ cells) and c-MYC expressing cells in tumors (n = 3 tumors per genotype). (**B**) Postmortem tumor volume at two weeks post-injection of fibrosarcoma cells (n = 6 control tumors; n = 5 *Mφ-c-Myc-KO* tumors). (**C**) Representative confocal images showing blood vessels (CD31^+^ endothelial cells). The graph shows quantification of CD31^+^ cells per selected tumor area using Imaris software (n = 3 tumors per genotype).

To assess whether c-*Myc* deletion in TAMs also affects the growth of other tumor types, we implanted 5×10^5^ JGA 95.1 murine fibrosarcoma cells in the flanks of *Mφ-c-Myc-KO* or control mice (two grafts/mouse). Animals were sacrificed two weeks post-grafting, and tumors were collected and analyzed. Confocal microscopy confirmed that CD68^+^ macrophages from tumors of control mice were positive for c-MYC, while c-MYC immunoreactivity was negligible in CD68^+^ cells from tumors grown in *Mφ-c-Myc-KO* mice (**Fig. 7A**). As in the B16 melanoma model, fibrosarcomas in *Mφ-c-Myc-KO* animals were significantly smaller than those isolated from control mice (**Fig. 7B**). Similarly, compared with controls, tumors from *Mφ-c-Myc-KO* mice exhibited a less developed vasculature (**Fig. 7C**).

## Discussion

TAMs play a major role in establishing a microenvironment able to support cancer development [12], but the molecular mechanisms controlling the differentiation and activity of these cells remain poorly characterized. It has been recently shown that *c-*MYC regulates *in vitro* human macrophage polarization and is expressed in macrophages infiltrating human tumors [28]. Possible clinical application of these findings first requires validation of the anti-tumor potential of targeting c-MYC function in TAMs in an animal model. Here we report the generation and characterization of *Mφ-c-Myc-KO* mice, in which *c-Myc* expression is inhibited in macrophages by using the loxP/LysM-Cre system [29]. Although *Mφ-c-Myc-KO* mice display no overt phenotypic changes under steady-state conditions, they are protected from tumor development in a graft model of B16 melanoma. Our studies demonstrate that TAMs isolated from tumors in *Mφ-c-Myc-KO* mice have reduced expression of the key pro-oncogenic factors VEGFA, HIF1α and MMP9. Remarkably, *Mφ-c-Myc-KO* mice also show defective tumor angiogenesis and reduced fibrosarcoma development, suggesting that c-MYC expression in TAMs contributes to the development of multiple tumor types.

Characterization of our model demonstrated that, under steady-state conditions, c-MYC expression is strongly reduced in peritoneal macrophages and BMDMs obtained from *Mφ-c-Myc-KO* mice, without affecting c-MYC expression in other tissues such as kidney, liver or testis. *Mφ-c-Myc-KO* mice exhibit normal BM hematopoiesis, since no differences in hematopoietic precursors could be detected by flow cytometry or clonogenic analysis (including LSKs, LT-HSCs, ST-HSCs, MMPs, MEPs, CMPs, GMPs and MPs). Consistent with the normal BM hematopoiesis, *Mφ-c-Myc-KO* mice have a normal content of immune cells in peripheral blood and normal levels of macrophage infiltration in immune organs.

To investigate tumor progression in control and *Mφ-c-Myc-KO* mice, we used the model of subcutaneous injection of B16 melanoma cells. *In vivo* luciferase bioluminescent assay and postmortem analysis demonstrated reduced tumor growth in *Mφ-c-Myc-KO* mice. This indicates that *c-Myc* depletion in macrophages critically affects their activation state, and consequently their efficiency in promoting tumor growth, consistent with recent reports that the differing abilities of tumors to proliferate and metastazise correlate with the differing pro-tumoral activities of associated TAMs [11], [13], [14].

Macrophages typically derive from circulating monoyctes and several studies have shown that the different TAM subtypes may originate from distinct monocyte precursors, including Ly6C-negative non-classical monocytes [49], [50] and Ly6C^high^MHCII^low^ classical monocytes [11], [51]. It has been suggested that Ly6C^high^MHCII^low^ monocytes that infiltrate tumors first give rise to Ly6C^high^MHCII^high^ TAMs, which then differentiate into Ly6C^low^MHCII^high^ TAMs [11]. We therefore analyzed the expression of Ly6C and MHC in TAMs isolated from tumors that developed in control and *Mφ-c-Myc-KO* mice two weeks after B16 melanoma cell injection. In control mice, we found a higher abundance of Ly6C^low^MHCII^high^ TAMs than Ly6C^high^MHCII^high^ TAMs (43.7±7.9 versus 17.1±5.3, p = 0.002, gated in CD11b^+^ cells). In contrast, TAMs in *Mφ-c-Myc-KO* mice displayed a delay in maturation, as indicated by similar levels of Ly6C^high^MHCII^high^ and Ly6C^low^MHCII^high^ subpopulations (28.4±11.5% Ly6C^high^MHCII^high^ versus 24.8±9.9% Ly6C^low^MHCII^high^, p = 0.13, gated in CD11b^+^ cells). Since c-MYC activity in macrophages contributes to the acquisition of the M2 phenotype [28], future studies are warranted to assess whether differences in the maturation state of macrophages resulting from *c-Myc* deficiency affects other pathophysiological processes that are regulated by M2 macrophages, such as parasite encapsulation, angiogenesis, wound healing, fetus rejection, allergy and asthma.

The higher abundance of a less mature TAM population in *Mφ-c-Myc-KO* tumors could explain the decreased tumor development, accounting for the impaired ability to promote cell proliferation, an inability to block immune anti-tumoral responses, and reduced production of pro-angiogenic molecules. In agreement with this, the proliferation of CD68 negative cells within tumors was diminished in *Mφ-c-Myc-KO* mice compared with those from control mice. Regarding the ability to counteract anti-tumoral responses, we found higher CD8^+^ T-lymphocyte infiltration in tumors from *Mφ-c-Myc-KO* mice than in tumors in control mice. This could be due, at least in part, to impaired capacity of *Mφ-c-Myc-KO* TAMs to inhibit CD8^+^ T-lymphocyte proliferation and to a higher level of CD11c expression in *Mφ-c-Myc-KO* TAMs, which may have increased ability to interact with and stimulate CD8^+^ T-lymphocytes.

Finally, we hypothesize that the TAM maturation state could also modulate tumor growth through the differential production of pro-tumoral molecules. Genetic deletion of *c-Myc* in the mouse leads to embryonic death, in part due to vasculogenesis defects caused by a failure of VEGF-dependent signaling [52], [53]. However, *c-Myc* deletion in endothelial cells alone does not prevent endothelial cell proliferation and vasculogenesis [54] because vascular remodeling in tumor development is modulated by TAMs [12]. Accordingly, we found that TAMs isolated from *Mφ-c-Myc-KO* tumors expressed reduced levels of the pro-angiogenic molecules HIF1α, VEGFA and MMP9. In line with *in vivo* findings, tumor-conditioned medium induced HIF1α, VEGFA and MMP9 expression in control but not in *Mφ-c-Myc-KO* BMDMs, and wound healing assays showed impaired proliferation and migration of endothelial cells treated with supernatants from *Mφ-c-Myc-KO* BMDMs exposed to tumor-conditioned medium compared with controls with intact *c-Myc*. We also found that this defect in pro-angiogenic molecules in tumors from *Mφ-c-Myc-KO* mice correlated with reduced development of new blood vessels, as assessed through the *postmortem* analysis of tumors by confocal microscopy and *in vivo* FMT using an Integrisense probe signal, which detects αvβ3 integrin positive endothelial cells, a hallmark of neovascularization [55]. Supporting the impaired angiogenesis observed in *postmortem* analysis, *in vivo* FMT analysis also revealed decreased MMPSense probe signal in tumors from *Mφ-c-Myc-KO* mice.

In summary, we have generated a new mouse model to investigate the role of c-MYC macrophage expression on tumor development. Characterization of this animal model in combination with *in vitro* studies identifies c-MYC as a positive regulator of the pro-tumoral program elicited by TAMs, which is essential for establishing a microenvironment that supports the growth of melanoma and fibrosarcoma. We believe that our results constitute a proof-of-concept that manipulating c-MYC expression has therapeutic potential for cancer treatment.

## References

[1] Cohen PE, Nishimura K, Zhu L, Pollard JW (1999) Macrophages: important accessory cells for reproductive function. J Leukoc Biol 66: 765–772. 10577508 10.1002/jlb.66.5.765

[2] Pollard JW (2009) Trophic macrophages in development and disease. Nat Rev Immunol 9: 259–270. 19282852 10.1038/nri2528 PMC3648866

[3] Allavena P, Sica A, Solinas G, Porta C, Mantovani A (2008) The inflammatory micro-environment in tumor progression: the role of tumor-associated macrophages. Crit Rev Oncol Hematol 66: 1–9. 17913510 10.1016/j.critrevonc.2007.07.004

[4] Balkwill F, Mantovani A (2010) Cancer and inflammation: implications for pharmacology and therapeutics. Clin Pharmacol Ther 87: 401–406. 20200512 10.1038/clpt.2009.312

[5] Hansen BD, Schmidt H, von der Maase H, Sjoegren P, Agger R, et al (2006) Tumour-associated macrophages are related to progression in patients with metastatic melanoma following interleukin-2 based immunotherapy. Acta Oncol 45: 400–405. 16760175 10.1080/02841860500471798

[6] Steidl C, Lee T, Shah SP, Farinha P, Han G, et al (2010) Tumor-associated macrophages and survival in classic Hodgkin's lymphoma. N Engl J Med 362: 875–885. 20220182 10.1056/NEJMoa0905680 PMC2897174

[7] Zhu X, Mulcahy LA, Mohammed RA, Lee AH, Franks HA, et al (2008) IL-17 expression by breast-cancer-associated macrophages: IL-17 promotes invasiveness of breast cancer cell lines. Breast Cancer Res 10: R95. 19014637 10.1186/bcr2195 PMC2656888

[8] Gordon S (2003) Alternative activation of macrophages. Nat Rev Immunol 3: 23–35. 12511873 10.1038/nri978

[9] Gordon S, Martinez FO (2010) Alternative activation of macrophages: mechanism and functions. Immunity 32: 593–604. 20510870 10.1016/j.immuni.2010.05.007

[10] Martinez FO, Gordon S, Locati M, Mantovani A (2006) Transcriptional profiling of the human monocyte-to-macrophage differentiation and polarization: new molecules and patterns of gene expression. J Immunol 177: 7303–7311. 17082649 10.4049/jimmunol.177.10.7303

[11] Movahedi K, Laoui D, Gysemans C, Baeten M, Stange G, et al (2010) Different tumor microenvironments contain functionally distinct subsets of macrophages derived from Ly6C(high) monocytes. Cancer Res 70: 5728–5739. 20570887 10.1158/0008-5472.CAN-09-4672

[12] Sica A, Larghi P, Mancino A, Rubino L, Porta C, et al (2008) Macrophage polarization in tumour progression. Semin Cancer Biol 18: 349–355. 18467122 10.1016/j.semcancer.2008.03.004

[13] Laoui D, Movahedi K, Van Overmeire E, Van den Bossche J, Schouppe E, et al (2012) Tumor-associated macrophages in breast cancer: distinct subsets, distinct functions. Int J Dev Biol 55: 861–867. 10.1387/ijdb.113371dl 22161841

[14] Laoui D, Van Overmeire E, Movahedi K, Van den Bossche J, Schouppe E, et al. 2011. Mononuclear phagocyte heterogeneity in cancer: different subsets and activation states reaching out at the tumor site. Immunobiology 216: 1192–1202. 21803441 10.1016/j.imbio.2011.06.007

[15] Meyer N, Penn LZ (2008) Reflecting on 25 years with MYC. Nat Rev Cancer 8: 976–990. 19029958 10.1038/nrc2231

[16] Blackwood EM, Eisenman RN (1991) Max: a helix-loop-helix zipper protein that forms a sequence-specific DNA-binding complex with Myc. Science 251: 1211–1217. 2006410 10.1126/science.2006410

[17] Lebel R, McDuff FO, Lavigne P, Grandbois M (2007) Direct visualization of the binding of c-Myc/Max heterodimeric b-HLH-LZ to E-box sequences on the hTERT promoter. Biochemistry 46: 10279–10286. 17705400 10.1021/bi700076m

[18] Levens DL (2003) Reconstructing MYC. Genes Dev 17: 1071–1077. 12730130 10.1101/gad.1095203

[19] Bowman T, Broome MA, Sinibaldi D, Wharton W, Pledger WJ, et al (2001) Stat3-mediated Myc expression is required for Src transformation and PDGF-induced mitogenesis. Proc Natl Acad Sci U S A 98: 7319–7324. 11404481 10.1073/pnas.131568898 PMC34666

[20] Dominguez-Caceres MA, Garcia-Martinez JM, Calcabrini A, Gonzalez L, Porque PG, et al (2004) Prolactin induces c-Myc expression and cell survival through activation of Src/Akt pathway in lymphoid cells. Oncogene 23: 7378–7390. 15286700 10.1038/sj.onc.1208002

[21] Klemsz MJ, Justement LB, Palmer E, Cambier JC (1989) Induction of c-fos and c-myc expression during B cell activation by IL-4 and immunoglobulin binding ligands. J Immunol 143: 1032–1039. 2787345

[22] Baena E, Ortiz M, Martinez AC, de Alboran IM (2007) c-Myc is essential for hematopoietic stem cell differentiation and regulates Lin(−)Sca-1(+)c-Kit(−) cell generation through p21. Exp Hematol 35: 1333–1343. 17637497 10.1016/j.exphem.2007.05.015

[23] Bianchi T, Gasser S, Trumpp A, MacDonald HR (2006) c-Myc acts downstream of IL-15 in the regulation of memory CD8 T-cell homeostasis. Blood 107: 3992–3999. 16449532 10.1182/blood-2005-09-3851

[24] de Alboran IM, Baena E, Martinez AC (2004) c-Myc-deficient B lymphocytes are resistant to spontaneous and induced cell death. Cell Death Differ 11: 61–68. 12970677 10.1038/sj.cdd.4401319

[25] Laurenti E, Varnum-Finney B, Wilson A, Ferrero I, Blanco-Bose WE, et al (2008) Hematopoietic stem cell function and survival depend on c-Myc and N-Myc activity. Cell Stem Cell 3: 611–624. 19041778 10.1016/j.stem.2008.09.005 PMC2635113

[26] Delgado MD, Leon J (2010) Myc roles in hematopoiesis and leukemia. Genes Cancer 1: 605–616. 21779460 10.1177/1947601910377495 PMC3092227

[27] Laurenti E, Wilson A, Trumpp A (2009) Myc's other life: stem cells and beyond. Curr Opin Cell Biol 21: 844–854. 19836223 10.1016/j.ceb.2009.09.006

[28] Pello OM, De Pizzol M, Mirolo M, Soucek L, Zammataro L, et al (2012) Role of c-Myc in alternative activation of human macrophages and tumor-associated macrophage biology. Blood 119: 411–421. 22067385 10.1182/blood-2011-02-339911

[29] Hume DA (2010) Applications of myeloid-specific promoters in transgenic mice support in vivo imaging and functional genomics but do not support the concept of distinct macrophage and dendritic cell lineages or roles in immunity. J Leukoc Biol 89: 525–538. 21169519 10.1189/jlb.0810472

[30] de Alboran IM, O'Hagan RC, Gartner F, Malynn B, Davidson L, et al (2001) Analysis of C-MYC function in normal cells via conditional gene-targeted mutation. Immunity 14: 45–55. 11163229 10.1016/s1074-7613(01)00088-7

[31] Tormo D, Checinska A, Alonso-Curbelo D, Perez-Guijarro E, Canon E, et al (2009) Targeted activation of innate immunity for therapeutic induction of autophagy and apoptosis in melanoma cells. Cancer Cell 16: 103–114. 19647221 10.1016/j.ccr.2009.07.004 PMC2851205

[32] Tiffen JC, Bailey CG, Ng C, Rasko JE, Holst J (2010) Luciferase expression and bioluminescence does not affect tumor cell growth in vitro or in vivo. Mol Cancer 9: 299. 21092230 10.1186/1476-4598-9-299 PMC3002927

[33] Efeyan A, Murga M, Martinez-Pastor B, Ortega-Molina A, Soria R, et al (2009) Limited role of murine ATM in oncogene-induced senescence and p53-dependent tumor suppression. PLoS One 4: e5475. 19421407 10.1371/journal.pone.0005475 PMC2675057

[34] Solinas G, Schiarea S, Liguori M, Fabbri M, Pesce S, et al (2010) Tumor-conditioned macrophages secrete migration-stimulating factor: a new marker for M2-polarization, influencing tumor cell motility. J Immunol 185: 642–652. 20530259 10.4049/jimmunol.1000413

[35] Redondo-Munoz J, Escobar-Diaz E, Samaniego R, Terol MJ, Garcia-Marco JA, et al (2006) MMP-9 in B-cell chronic lymphocytic leukemia is up-regulated by alpha4beta1 integrin or CXCR4 engagement via distinct signaling pathways, localizes to podosomes, and is involved in cell invasion and migration. Blood 108: 3143–3151. 16840734 10.1182/blood-2006-03-007294

[36] Lopez-Rivera E, Lizarbe TR, Martinez-Moreno M, Lopez-Novoa JM, Rodriguez-Barbero A, et al (2005) Matrix metalloproteinase 13 mediates nitric oxide activation of endothelial cell migration. Proc Natl Acad Sci U S A 102: 3685–3690. 15728377 10.1073/pnas.0408217102 PMC553299

[37] Pixley FJ, Stanley ER (2004) CSF-1 regulation of the wandering macrophage: complexity in action. Trends Cell Biol 14: 628–638. 15519852 10.1016/j.tcb.2004.09.016

[38] Arpa L, Valledor AF, Lloberas J, Celada A (2009) IL-4 blocks M-CSF-dependent macrophage proliferation by inducing p21Waf1 in a STAT6-dependent way. Eur J Immunol 39: 514–526. 19130475 10.1002/eji.200838283

[39] Valledor AF, Arpa L, Sanchez-Tillo E, Comalada M, Casals C, et al (2008) IFN-{gamma}-mediated inhibition of MAPK phosphatase expression results in prolonged MAPK activity in response to M-CSF and inhibition of proliferation. Blood 112: 3274–3282. 18682602 10.1182/blood-2007-11-123604

[40] Adolfsson J, Borge OJ, Bryder D, Theilgaard-Monch K, Astrand-Grundstrom I, et al (2001) Upregulation of Flt3 expression within the bone marrow Lin(−)Sca1(+)c-kit(+) stem cell compartment is accompanied by loss of self-renewal capacity. Immunity 15: 659–669. 11672547 10.1016/s1074-7613(01)00220-5

[41] Iwasaki H, Akashi K (2007) Myeloid lineage commitment from the hematopoietic stem cell. Immunity 26: 726–740. 17582345 10.1016/j.immuni.2007.06.004

[42] Fogg DK, Sibon C, Miled C, Jung S, Aucouturier P, et al (2006) A clonogenic bone marrow progenitor specific for macrophages and dendritic cells. Science 311: 83–87. 16322423 10.1126/science.1117729

[43] Geissmann F, Gordon S, Hume DA, Mowat AM, Randolph GJ (2010) Unravelling mononuclear phagocyte heterogeneity. Nat Rev Immunol 10: 453–460. 20467425 10.1038/nri2784 PMC3032581

[44] Gordon S (1995) The macrophage. Bioessays 17: 977–986. 8526892 10.1002/bies.950171111

[45] Dirkx AE, Oude Egbrink MG, Wagstaff J, Griffioen AW (2006) Monocyte/macrophage infiltration in tumors: modulators of angiogenesis. J Leukoc Biol 80: 1183–1196. 16997855 10.1189/jlb.0905495

[46] Montet X, Figueiredo JL, Alencar H, Ntziachristos V, Mahmood U, et al (2007) Tomographic fluorescence imaging of tumor vascular volume in mice. Radiology 242: 751–758. 17325064 10.1148/radiol.2423052065

[47] Sipkins DA, Cheresh DA, Kazemi MR, Nevin LM, Bednarski MD, et al (1998) Detection of tumor angiogenesis in vivo by alphaVbeta3-targeted magnetic resonance imaging. Nat Med 4: 623–626. 9585240 10.1038/nm0598-623

[48] Osthus RC, Shim H, Kim S, Li Q, Reddy R, et al (2000) Deregulation of glucose transporter 1 and glycolytic gene expression by c-Myc. J Biol Chem 275: 21797–21800. 10823814 10.1074/jbc.C000023200

[49] MacDonald KP, Palmer JS, Cronau S, Seppanen E, Olver S, et al (2010) An antibody against the colony-stimulating factor 1 receptor depletes the resident subset of monocytes and tissue- and tumor-associated macrophages but does not inhibit inflammation. Blood 116: 3955–3963. 20682855 10.1182/blood-2010-02-266296

[50] Pucci F, Venneri MA, Biziato D, Nonis A, Moi D, et al (2009) A distinguishing gene signature shared by tumor-infiltrating Tie2-expressing monocytes, blood "resident" monocytes, and embryonic macrophages suggests common functions and developmental relationships. Blood 114: 901–914. 19383967 10.1182/blood-2009-01-200931

[51] Pahler JC, Tazzyman S, Erez N, Chen YY, Murdoch C, et al (2008) Plasticity in tumor-promoting inflammation: impairment of macrophage recruitment evokes a compensatory neutrophil response. Neoplasia 10: 329–340. 18392134 10.1593/neo.07871 PMC2288539

[52] Baudino TA, McKay C, Pendeville-Samain H, Nilsson JA, Maclean KH, et al (2002) c-Myc is essential for vasculogenesis and angiogenesis during development and tumor progression. Genes Dev 16: 2530–2543. 12368264 10.1101/gad.1024602 PMC187450

[53] Mezquita P, Parghi SS, Brandvold KA, Ruddell A (2005) Myc regulates VEGF production in B cells by stimulating initiation of VEGF mRNA translation. Oncogene 24: 889–901. 15580293 10.1038/sj.onc.1208251

[54] He C, Hu H, Braren R, Fong SY, Trumpp A, et al (2008) c-myc in the hematopoietic lineage is crucial for its angiogenic function in the mouse embryo. Development 135: 2467–2477. 18550710 10.1242/dev.020131 PMC2597486

[55] Snoeks TJ, Lowik CW, Kaijzel EL (2010) 'In vivo' optical approaches to angiogenesis imaging. Angiogenesis 13: 135–147. 20449766 10.1007/s10456-010-9168-y PMC2911541

